# Rabeprazole suppressed gastric intestinal metaplasia through activation of GPX4-mediated ferroptosis

**DOI:** 10.3389/fphar.2024.1409001

**Published:** 2024-11-07

**Authors:** Jing Xie, Xinhua Liang, Fangfang Xie, Canxin Huang, Zijun Lin, Shuping Xie, Fangying Yang, Fengfeng Zheng, Lanlan Geng, Wanfu Xu, Sitang Gong, Li Xiang

**Affiliations:** ^1^ Department of Gastroenterology, Guangzhou Women and Children’s Medical Center, Guangzhou Medical University, Guangzhou, China; ^2^ School of Second Clinical Medicine, Guangzhou Medical University, Guangzhou, China; ^3^ Guangzhou Institute of Pediatrics, Guangzhou Women and Children’s Medical Center, Guangzhou Medical University, Guangzhou, China; ^4^ Department of Infectious Diseases, The Affiliate Hospital of Putian University, Putian, China

**Keywords:** *H. pylori*, gastric intestinal metaplasia, rabeprazole, ferroptosis, CREB

## Abstract

**Background:**

Gastric intestinal metaplasia is a common pathological feature in patients with *Helicobacter pylori* (*H. pylori*) infection. Rabeprazole was widely used as the first-line regimen for *H. pylori* infectious treatment. The objective of this study is to explore the mechanism of rabeprazole in gastric intestinal metaplasia treatment.

**Methods:**

Real-time PCR, Western blotting (WB) and ROS analysis were conducted to confirm that rabeprazole could induce ferroptosis to suppress gastric intestinal metaplasia. Cellular fraction, luciferase and chromatin immunoprecipitation (ChIP) were used to identify the mechanism underlying rabeprazole modulated ferroptosis.

**Results:**

Herein, we found rabeprazole treatment led to inhibit CDX2 and MUC2 expression, alleviating gastric intestinal metaplasia, which was attributed to enhanced ferroptosis characterized by decreased GPX4 expression. Inhibition of ferroptosis by ferrostatin-1 (Fer-1) could reverse decreased CDX2 and MUC2 expression caused by rabeprazole. Mechanically, Rabeprazole could inhibit CREB phosphorylation and nuclear translocation, which further decreased the binding of CREB to GPX4 promoter, reducing GPX4 transactivity. Moreover, endogenous PKA interacted with CREB, and this interaction was drastically destroyed in response to rabeprazole treatment. Most importantly, enhanced ferroptosis was observed in *H. pylori-*infected gastric intestinal metaplasia in comparison to HC control.

**Conclusion:**

These findings suggested that rabeprazole induced ferroptosis to reduce CDX2 expression in gastric epithelial cells through PKA/CREB cascade signaling, implying that targeting ferroptosis could be a promising strategy in improving gastric intestinal metaplasia during *H. pylori*-infected patients.

## Introduction

*Helicobacter pylori* (*H. pylori*) is well known as a risk factor for gastritis and gastric intestinal metaplasia (IM) (Scida et al., 2018; Zheng et al., 2022), which has been regarded as a precancerous lesion in the *H. pylori*-induced metaplasia-dysplasia-carcinoma sequence characterized by enhanced caudal-related homeobox 2 (CDX2) and/or mucin 2 (MUC2) expression (Chen et al., 2021; Chen et al., 2020). Currently, a standard triple therapy consisted of Proton pump inhibitors (PPIs) and antibiotics was widely used as the first-line regimen for *H. pylori* infectious treatment. In addition to inhibition of gastric acid secretion, PPI was gained more and more attention due to the novel biological function. For instance, PPI, including Omeprazole and Lansoprazole, was showed to protect against intestinal injury and alleviate neutrophil-dependent gastric mucosal inflammation (Handa et al., 2006; Ichikawa et al., 2004). Moreover, Rabeprazole was reported to suppress cell pyroptosis and destroy gastric epithelial barrier function through Forkhead Box F1 (FOXF1)/signal transducer and activator of transcription 3 (STAT3)-mediated ZO-1 expression (Yang et al., 2023; Xie et al., 2021), and Rabeprazole has ability to inhibit STAT3-mediated glycolysis, leading to reduce cell proliferation (Zhou et al., 2021). However, further work has found that the long-term PPI use destroyed intestinal tight junction barrier to exaggerate experimental colitis (Nighot et al., 2023) and exhibited a significantly higher risk observed for intestinal metaplasia in dose-dependent manner (Lv F. et al., 2023; Snir et al., 2021). Neverless, the mechanism and effect of Rabeprazole in *H. pylori-*induced intestinal metaplasia remained unclear completely.

Ferroptosis, an iron- and reactive oxygen species (ROS)-dependent programmed cell death, is characterized by downregulation of the antioxidant peroxidase glutathione peroxidase 4 (GPX4), which further regulated ROS clearance, followed by the accumulation of lipid peroxidation products or inhibition of the system Xc-, a cystine glutamate antiporter composed of SLC7A11 (system Xc-) (Wu et al., 2023; Pan et al., 2023). Recently, ferroptosis has been documented to play a critical role in *H. pylori-*related disease, including gastric cancer (Zhu et al., 2023; Ni et al., 2021). For instance, miR-375 decreased GC cell stemness through targeting ferroptosis. What’s more, inhibition of cAMP response element-binding protein (CREB)-mediated ferroptosis could trigger CDX2 expression in intestinal epithelial cells to promote mucosal healing, suggesting ferroptosis may be a critical event for gastric intestinal metaplasia during *H. pylori* infection. In addition, activation of CREB and nuclear factor erythroid 2-related factor 2 (NRF2) signaling were showed to suppress ferroptosis through inducing GPX4 and SLC7A11 expression (Pan et al., 2023; Lv et al., 2023). Further bioinformatics analysis has revealed that 17 differently expressed ferroptosis-related genes was selected, NOS2 and HMOX1 was confirmed the validity and robustness of HMOX1 and NOS2 genes in diagnose IM, which could be as IM biomarkers, and high expression levels of HMOX1 gene in patients with gastric cancer was identified to have shorter overall survival (Song et al., 2023). However, the mechanism of ferroptosis alteration in IM remained unknown.

In this work, we tried to explore that whether rabeprazole alleviated gastric intestinal metaplasia through ferroptosis, which could further extend the novel mechanism of rabeprazole modulated gastric intestinal metaplasia, and targeting ferroptosis could serve as a potential therapy strategy to alleviate gastric intestinal metaplasia.

## Methods

### Reagents and antibodies

Dulbecco’s modified Eagle’s medium (DMEM, C11995500BT), and fetal bovine serum (FBS, 10099141C) were purchased from Life Technologies (Carlsbad, CA, United States); Phenylmethanesulfonyl fluoride (PMSF, P0100) and protease inhibitor cocktail (PIC, P6730) were from Solarbio (Beijing, China); Ratio-immunoprecipitation assay (RIPA) lysis buffer (P0013), Nuclear and Cytoplasmic Protein Extraction Kit (P0028), antifade mounting medium with DAPI(P0131), ROS detection kit (S0033S) and bicinchoninic acid (BCA) protein assay kit (P0012) were from Beyotime Biotechnology (Shanghai, China); EZ-press RNA Purification Kit (B0004D) was from EZBioscience (Shanghai, China). TB Green^®^ Premix Ex Taq™ II (Tli RNaseH Plus) (RR820B) and PrimeScript™ RT Master Mix (Perfect Real Time) (RR036A) were obtained from TaKaRa (Dalian, China). Dual-Luciferase Reporter Assay was purchased from Promega (E1910, Madison, WI, United States). SimpleChIP^®^ Plus Enzymatic Chromatin IP Kit (Magnetic Beads) (9005) was purchased from Cell Signaling Technology (Danvers, MA, United States). Rabeprazole (HY-B0656) and Ferrostatin-1 (HY-100579) were from MedChemExpress (NJ, United States). All ultrapure reagents were from Biosharp (Guangzhou, China). Antibody against CDX2 monoclonal antibody (60243-1-Ig, Proteintech), SLC7A11/xCT polyclonal antibody (26864-1-AP, Proteintech), GPX4 monoclonal antibody (67763-1-Ig, Proteintech), Lamin A/C recombinant antibody (81042-1-RR, Proteintech), CREB1 Monoclonal antibody (67927-1-Ig, Proteintech) and phospho-CREB1 (Ser133) Polyclonal antibody (28792-1-AP, Proteintech) were from Proteintech (Wuhan, China). Anti-MUC2 antibody (ab272692, Abcam) was from Abcam (Cambridge, UK). PKA C-alpha (PRKACA) rabbit pAb (A0798, Abclonal) was purchased from Abclonal (Wuhan, China). β-tubulin (MG7) mouse monoclonal antibody (RM 2003, Ray) was purchased from Ray biotech (Beijing, China). Peroxidase-conjugated AffiniPure Goat Anti-Rabbit IgG (H + L) (111-035-003) and Peroxidase-conjugated AffiniPure Goat Anti-Mouse IgG (H + L) (115-035-003) were purchased from Jackson ImmunoResearch (Jackson, United States), Alexa 594-conjugated secondary antibodies (RS3608) was from immunoway Research (Plano, United States).

### Cell culture, treatment, and transfection

As described in our previous study (Liang et al., 2023), HEK293, AGS, and BGC823 cells were purchased from American Type Culture Collection (ATCC) and cultured in DMEM containing 10% FBS. For treatment, gastric epithelial cells were used in the whole study and treated with rabeprazole (100 uM), or rabeprazole combined with ferrostatin-1(Fer-1, 10 uM) for 48 h. For transfection, plasmid or siRNA was transfected into cell using lipofectamine 3000 according to the manufacturer’s protocol. CREB plasmid was from Youbio (Changshang, China).

### ROS detection level

The ROS level was measured using DCFH-DA staining according to our previous work (Pan et al., 2023). Briefly, the cells were seeded at a density of 10^5^/mL for 24 h, subsequently following by treatment with or without rabeprazole for 48 h, after washing with PBS, DCFH-DA solution was added to incubate for another 20 min at 37°C and 5%CO_2_. The ROS level was visualized and analyzed by flow cytometry.

### RNA extraction and qPCR analysis

As described in our pervious study (Zhang et al., 2019), the total RNA was extracted using EZ-press RNA Purification Kit and reversed transcript into cDNA. Quantitative PCR (qPCR) were performed according to the manufacturer’s instructions. Primer sequences used in this study were listed as followed: CDX2: forward:5′-CTCGGCAGCCAAGTGAAAACCA-3′, and reverse: 5′-GCTTTCCTCCGGATGGTGATGTA-3′(Sun et al., 2017); MUC2: forward:5’-CAGGATGGCGCCTTCTGCTA-3’, and reverse:5’-ATGCTGCTCCAAGCTGAGGT-3’ (Li et al., 2020); GPX4 forward, 5’-GAG​GCA​AGA​CCG​AAG​TAA​ACT​AC-3’; reverse, 5′-CCG​AAC​TGG​TTA​CAC​GGG​AA-3′; SLC7A11 forward,5’-ACGGTGGTGTGTTTGCTGTCTC-3’; reverse, 5’-GCT​GGT​AGA​GGA​GTG​TGC​TTG​C-3’ (Pan et al., 2023); UBC: forward:5’-ATTTGGGTCGCGGTTCTTG-3’ and reverse: 5’-TGC​CTT​GAC​ATT​CTC​GAT​GGT-3’ (Zou et al., 2012).

### Subcellular fractionation and immunoblotting analysis

Total protein was extracted with RIPA lysis buffer. Nuclear and cytosolic proteins were isolated by nuclear extraction kit and determined using BCA protein assay kit. Western blotting was performed in previous study (Wang et al., 2021). Briefly, proteins were subjected to SDS-PAGE and transferred into nitrocellulose transfer membrane, following by blocking with 5% slim milk in PBS/0.05% tween (PBST) for 1 h. The primary antibodies were added to incubate indicated band overnight at 4°C, followed by incubation with secondary antibodies (Jackson immunoresearch, UK) for 1 h at room temperature. Proteins were imaged using an enhanced chemiluminescence (Perkin Elmer).

### CCK8 analysis

As described in our previous work (Xu et al., 2017), cells were seeded into 96-well plate overnight, after treatment for 48 h, each well was induced with fresh medium contained 10 μl CCK-8 in 90 μl of culture medium. The cells were incubated for 1 h at 37 °C, and absorbance were measured at 450 nm.

### Immunofluorescence

Immunofluorescence was performed as described in our work (Zhou et al., 2021; Pan et al., 2023). The gastric epithelial cells were seeded at a density of 0.5*10^5^/mL in 6-well plates placed glass coverslip overnight. After rabeprazole treatment for 1 h, cells were fixed with 4% paraformaldehyde for 15 min and permeabilized using 0.5% Triton X-100 for 20 min and then blocked in 10% goat serum for 30 min. The primary antibody targeted CREB was added to incubate overnight at 4 °C. For tissue slides were deparaffinized, incubated with blocking buffer (PBS with 5% normal donkey or goat serum and 0.3% Triton X-100) at room temperature for 1 h, and stained with primary antibodies overnight in a wet chamber at 4°C in the dark, and then incubation was required for another 1 h with Alex-488/594 conjugated secondary antibodies at room temperature. The coverslips were mounted onto glass slides with prolong gold reagent after staining the nuclei with 4′,6-diamidino-2-phenylindole (DAPI). Stained cells were visualized using a laser scanning confocal fluorescent microscope.

### Immunoprecipitation

As described in our pervious study (Pan et al., 2023), after treatment with or without rabeprazole for 1 h, the total cells were harvested with RIPA for 15 min lysis were incubated with anti-PKA or nonspecific immunoglobulin (IgG) overnight at 4°C. The beads were washed with ice cold RIPA buffer and added to incubate with lysate for another 2 h. Elution was lysis with 2 × SDS-PAGE protein sample buffer and boiled at 95 °C for 10 min. Protein expression was detected using western blots.

### Luciferase assay

As described in our previous work (Pan et al., 2023; Li et al., 2020), luciferase reporter plasmid and internal control plasmid were co-transfected into cells using lipofectamine 3000 according to manufacturer’s instruction. 12 h after transfection, the cell was treated with or without rabeprazole for another 48 h, and firefly and renilla luciferase value were measured using the dual‐luciferase reporter assay system (Promega).

### Chromatin immunoprecipitation

As described in our pervious study (Pan et al., 2023; Xu et al., 2018; Xu et al., 2019), ChIP assay was performed according to the protocol of Sample ChIP(R) Plus Kit (Magnetic Bead) (Cell Signal Technology, 9005) with anti-CREB or negative control anti‐IgG. The precipitated DNAs was analyzed and quantified by using real‐time PCR analysis with following primers (Wang et al., 2021): GPX4 forward: 5’-AAG​CGA​GCA​TGC​GCA​GTC​GCC​AA-3’; reverse: 5’-GGA​CGC​GCG​TCG​GCT​TTC​CGC​G-3’.

### Statistical analysis

All statistical analyses were conducted using Prism 9. Each experiment was performed for three biological replicates. One-Sample t-test was used to analyze the difference in qPCR assay, the band intensity was quantified by one sample t-test (control group was nornmailzed as 1) or one way-ANOVA. The luciferase activity was analyzed by two sample t-test or two-ANOVA with multiple comparisons, followed by Bonferroni *post hoc* test for significance. *p*-value less than 0.05 was taken to indicate statistical significance.

## Results

### Rabeprazole suppressed CDX2 expression

**FIGURE 1:**
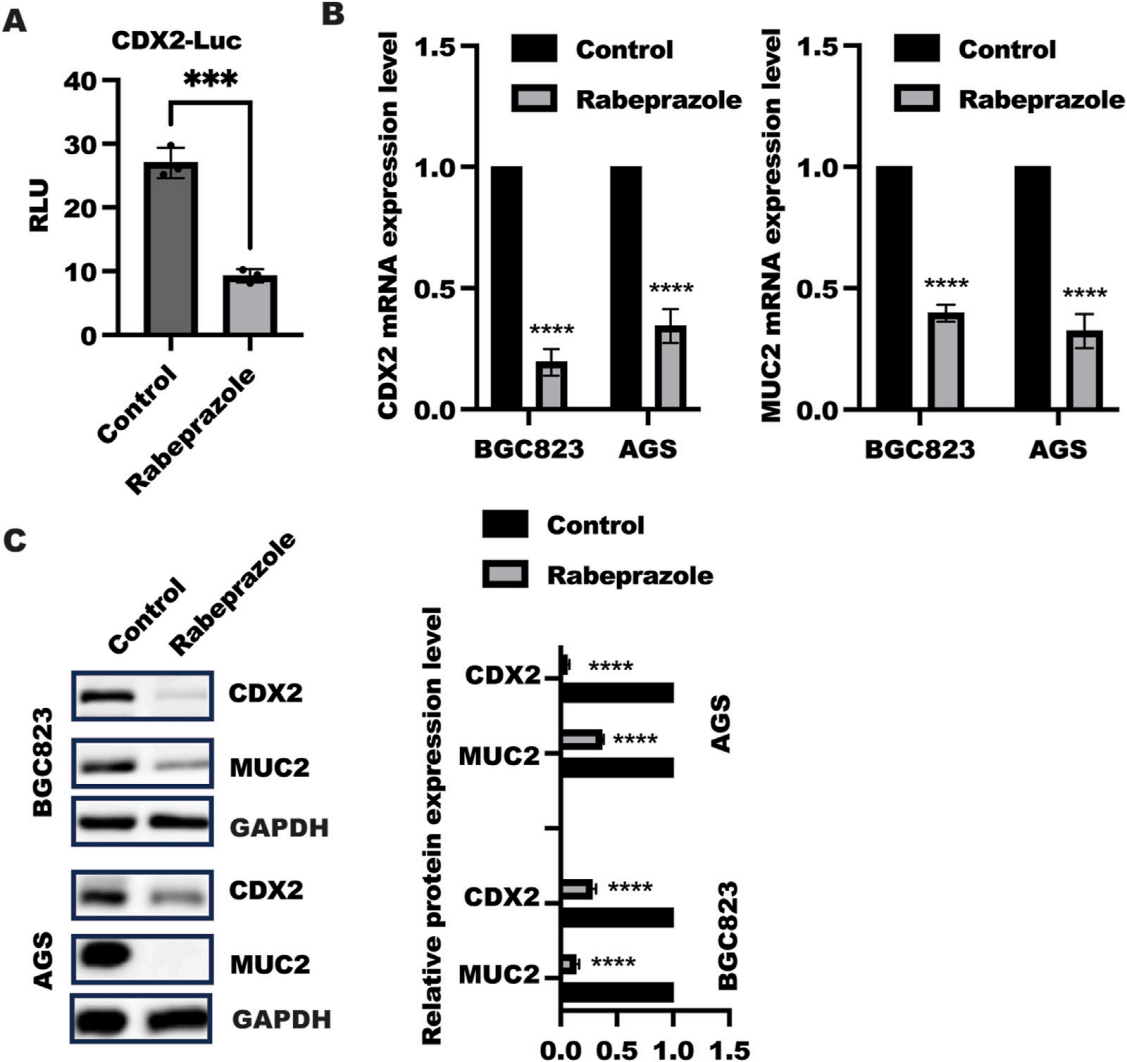
Rabeprazole suppressed gastric intestinal metaplasia. **(A)** HEK293 cells were transfected with reporter gene containing CDX2 promoter and renilla plasmid for 12 h, following by treatment with or without rabeprazole for another48 h, the CDX2 transactivation was determined and analyzed using two sample t-test. Data was exhibited as mean ± s.d of three independent experiments, ***p < 0.001. **(B)** After treatment with or without rabeprazole for 48 h in BGC823 and AGS cells, the whole RNA was extracted to detect CDX2 (*left panel*) and MUC2 (*right panel*) mRNA level, data was displayed as mean ± s.d of three independent experiments, one sample t-test was employed to determine the significance. ****p < 0.0001; **(C)** After cell adhesion overnight, BGC823 and AGS cells were starved with serum-free medium for 16 h and treated with or without rabeprazole (100uM) for another 48 h. The whole cell lysate was collected and detected indicated protein expression by immunoblotting, band intensity was analyzed and quantified using two sample t-test, Data was exhibited as mean ± s.d of three independent experiments, ****p < 0.0001.

To explore the possible role of rabeprazole in gastric intestinal metaplasia, we treated BGC823 and AGS cells with rabeprazole for 48 h to detect relative protein expression involved in gastric intestinal metaplasia, including CDX2 and its target gene MUC2. As shown in Figure 1A, the CDX2 transactivation was largely decreased in HEK293 transfected with reporter gene containing CDX2 promoter and renilla plasmid after rabeprazole treatment. What’s more, the results showed that a significant downregulation of CDX2 and MUC2 mRNA level was found in BGC823 and AGS cells received with rabeprazole treatment (Figure 1B), in line with this, the reduced CDX2 and MUC2 protein expression were observed in BGC823 and AGS cells after rabeprazole treatment (Figure 1C). Taken together, these findings suggested rabeprazole has an influence in alleviating gastric intestinal metaplasia.

### Rabeprazole induced ferroptosis to alleviate gastric intestinal metaplasia

**FIGURE 2:**
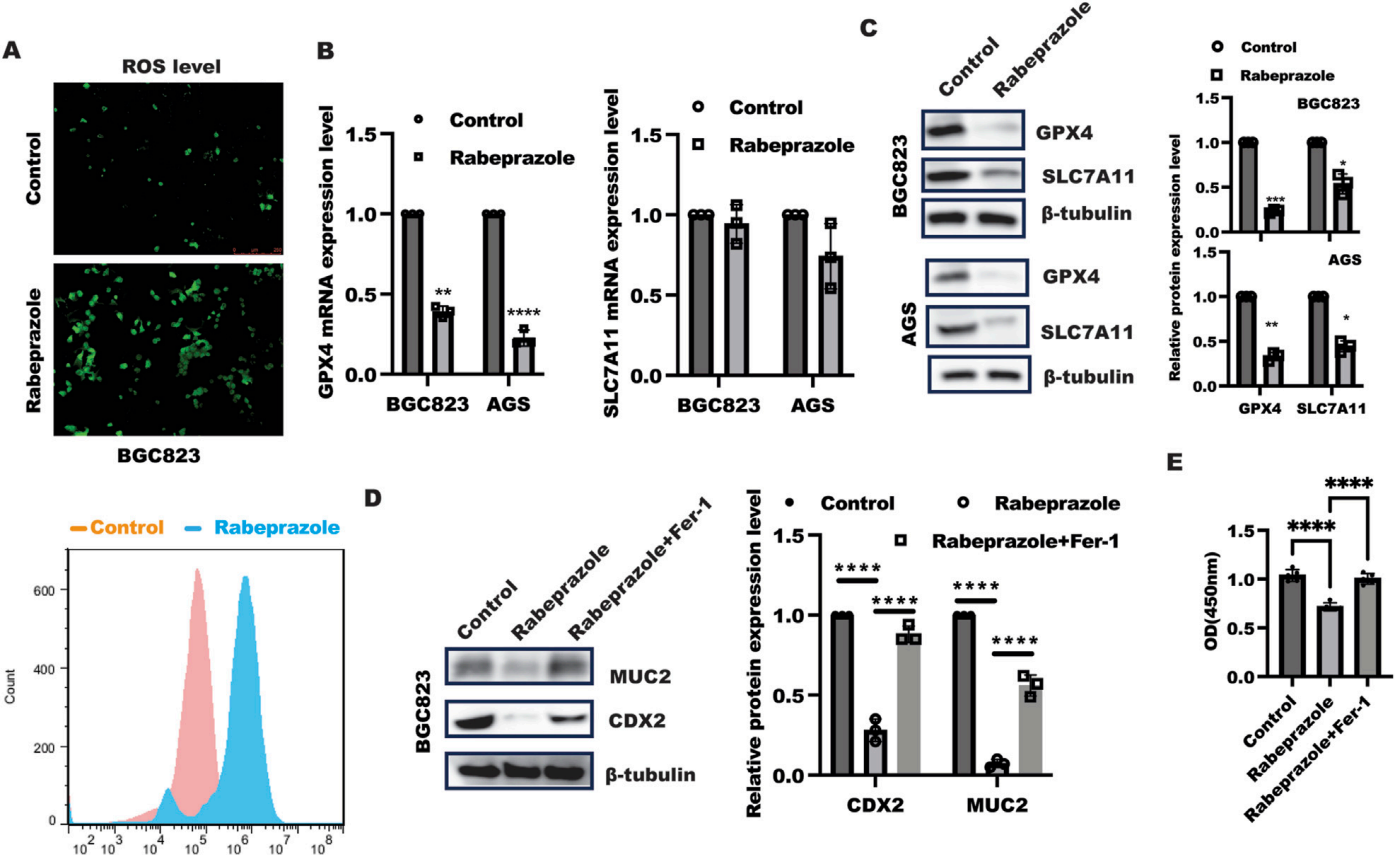
Rabeprazole modulated gastric intestinal metaplasia in dependent of ferroptosis. **(A)** BGC823 cells were digested and re-seeded into a 24-well plate and grown to 80% confluence, after serum-sstarvation overnight, BGC823 cells were treated with or without rabeprazole (100uM) for 48 h, the ROS level was detected according to the manufacter’s instruction and captured under fluorescent microscope (upper panel) as well as FCM analysis (bottom panel), bar: 250 um. **(B)** BGC823 and AGS cells were cultured without FBS overnight and incubated with or without rabeprazole (100uM) for another 48 h, real-time PCR was utilized to test indicated genes expression, control group was normalized as 1, data was exhibited as mean ± s.d of three independent experiments, one sample t-test was used to determine the significance. **p < 0.01, ****p < 0.0001. **(C)** BGC823 and AGS cells was treated as described in B, immunoblotting was utilized to analyze GPX4 and SLC7A11 protein expression. Control group was normalized as 1, data was exhibited as mean ± s.d of three independent experiments, one sample t-test was used to determine the significance. *p < 0.05, **p < 0.01, ****p < 0.0001. **(D)** After serum-starved culture overnight, BGC823 cells were treated with Ferrostatin-1 (Fer-1, 10 uM) for 1 h, subsequently followed by treatment with rabeprazole for another 48 h. The total protein was harvested to test MUC2 and CDX2 expression by immunoblotting. the band intensity was measured and quantified by one-ANOVA with multiple comparisons, followed by Bonferroni *post hoc* test for significance, control group was normalized as 1, data was exhibited as mean ± s.d of three independent experiments, ****p < 0.0001. **(E)** CCK8 analysis of cell viability in BGC823 cells treated as indicated for 48 h, data was exhibited as mean ± s.d of five independent experiments, one-ANOVA with multiple comparisons, followed by Bonferroni *post hoc* test for significance, ****p < 0.0001.

The above findings implied rabeprazole could suppress gastric intestinal metaplasia, however, how rabeprazole regulated gastric intestinal metaplasia remained unknown. Our previous work has demonstrated inhibition of ferroptosis by GPX4 overexpression in intestinal epithelial cells could enhance CDX2 expression (Pan et al., 2023), which focused us to verify the possibility that impaired gastric intestinal metaplasia caused by rabeprazole was attributed to enhanced ferroptosis. As shown in Figure 2A, rabeprazole treatment in BGC823 and AGS cells led to enhance ROS level, further work showed that GPX4, not SLC7A11, was markedly reduced at mRNA and protein level in BGC823 and AGS cells in response to rabeprazole treatment (Figures 2B, C), sugesting rabeprazole treatment led to ferroptosis. To further confirm inhibition of ferroptosis could reverse the inhibitory effect of rabeprazole on CDX2 expression, we used ferroptosis inhibitor ferrostatin-1 (Fer) (10 μM) combined with rabeprazole to confirm the hypothesis. As expected, ferrostatin-1 treatment in BGC823 cells led to a reversed effect of rabeprazole on CDX2 and MUC2 expression (Figure 2D). moreover. Inhibition of ferroptosis by Fer could reverse the inhibitory effect of rabeprazole on cell viability (Figure 2E). These findings suggested rabeprazole suppressed CDX2 through activation of ferroptosis.

### Rabeprazole-modulated GPX4 expression required CREB

**FIGURE 3:**
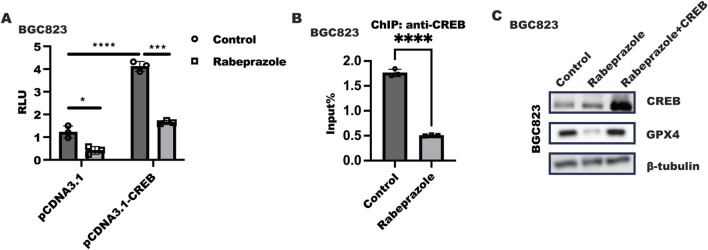
Rabeprazole suppressed GPX4 expression in dependent of CREB. **(A)** Luciferase reporter containing GPX4-promoter and renilla plasmid (pGL4.74) combined with pCDNA3.1 or pCDNA3.1-CREB plasmid were transfected into BGC823 cells for 24 h, after serum starvation overnight, cells were treated with or without rabeprazole treatment for another 48 h. The relative luminescence units (RLU) were detected using duo-lite luciferase assay system. Data was exhibited as mean ± s.d of three independent experiments and quantified by two-ANOVA with multiple comparisons, followed by Bonferroni *post hoc* test for significance. n = 3, ****p < 0.0001, ***p < 0.001, *p < 0.05. **(B)** After serum starvation overnight, BGC823 cells were treated with or without rabeprazole for 1 h, ChIP analysis was performed to analyze the effect of rabeprazole on CREB binds to GPX4 promoter, data was displayed as mean ± s.d of three independent experiments and analyzed by two sample t-test for significance. ****p < 0.0001. **(C)** CREB plasmid was transfected into BGC823 cells for 12 h and serum-starved for 12 h, followed by treatment with or without rabeprazole for another 24 h, the total protein was collected to test indicated protein expression level.

Next, we sought to explore how rabeprazole regulated GPX4 expression. Because of CREB has been reported to initiate GPX4 transactivation, luciferase and ChIP were utilized to confirm the hypothesis that rabeprazole regulated GPX4 expression in dependent of CREB. As illustrated in Figure 3A, GPX4 transactivation was strongly ablated in BGC823 cells after receiving rabeprazole treatment, while ectopic expression of CREB could largely block the inhibitory effect of rabeprazole on GPX4 transactivation. What’s more, ChIP analysis showed that rabeprazole treatment in BGC823 cells could decrease the binding ability of CREB to GPX4 promoter (Figure 3B). These results indicated that CREB has a significant role in rabeprazole-mediated GPX4 transactivation. Most importantly, overexpression of CREB could overcome the effect of rabeprazole on GPX4 and CDX2 expression (Figure 3C). Taken together, these results implied that CREB has an important role in rabeprazole-mediated GPX4 expression.

### Rabeprazole inhibited PKA/CREB cascade signaling to decrease CREB nuclear translocation

**FIGURE 4:**
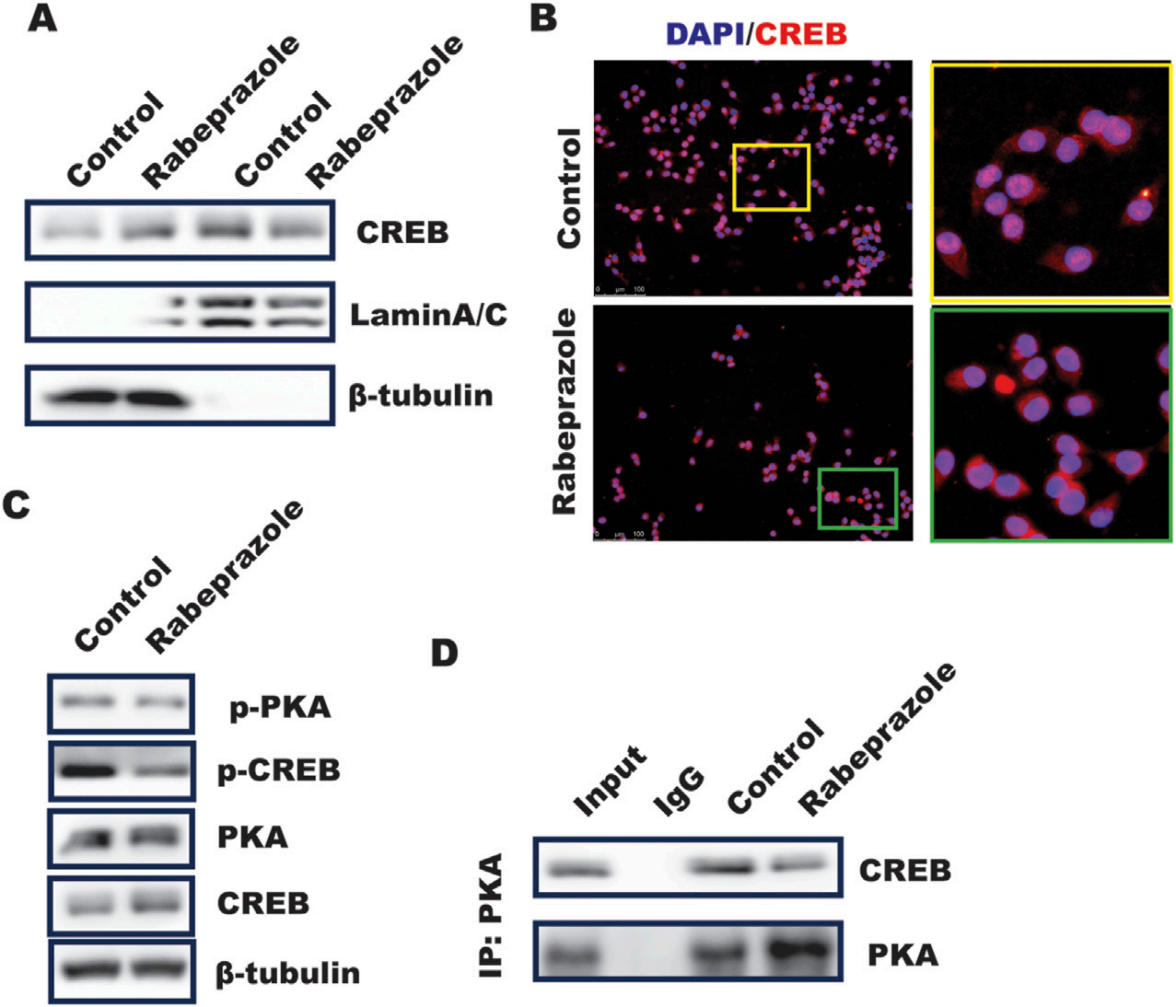
Rabeprazole suppressed CREB nuclear translocation and phosphorylation. **(A)** Cell fraction was isolated using nuclear extraction kit according to the instruction to analyze the effect of rabeprazole on cytosolic and nuclear CREB level, laminA/C and b-actin was taken as nucleus and cytosolic internal control, respectively. **(B)** Immunofluorescence analysis of CREB location in BGC823 cells received with or without rabeprazole treatment for 1 h **(C)** the whole cell lysate was collected from BGC823 cells treated with or without rabeprazole for 1 h and subjected to WB analysis to determine indicated protein expression. **(D)** BGC823 cells were treated with or without rabeprazole for 1 h, the whole cell lysate was immunoprecipitated with PKA, the immunoblotting was used to detect CREB expression.

The above results implied phosphorylation of CREB mediated by rabeprazole is critical for GPX4 expression, however, the mechanism through which rabeprazole modulated CREB activation remained to be addressed. Our previous work has revealed that inhibition of PDE4 by roflumilast could trigger PKA/CREB pathway to activate GPX4 transcription (Pan et al., 2023), which attracted us to explore the function of rabeprazole on PKA/CREB signaling. Phosphorylation of CREB at Ser133 could induce itself nuclear translocation, which further initiated CREB-dependent genes transcription (Silva-Garcia et al., 2018). As illustrated in Figure 4A, a subcellular fractionation analysis demonstrated that CREB nuclear level was decreased in BGC823 cells in response to rabeprazole treatment. In line with this, IF analysis also found that rabeprazole stimulation led to a significant downregulation of CREB nuclear translocation (Figure 4B), which was attributed to phosphorylation of PKA/CREB was reduced in BGC823 cells caused by rabeprazole treatment (Figure 4C). Further analysis showed that endogenous PKA interacted with CREB, and this interaction was destroyed in response to rabeprazole treatment (Figure 4D). Taken together, these results suggested that rabeprazole destroyed PKA/CREB complex to inhibit CREB activation and nuclear translocation, leading to decrease GPX4 transactivation.

### Decreased ferroptosis was observed in patients with gastric intestinal metaplasia

**FIGURE 5:**
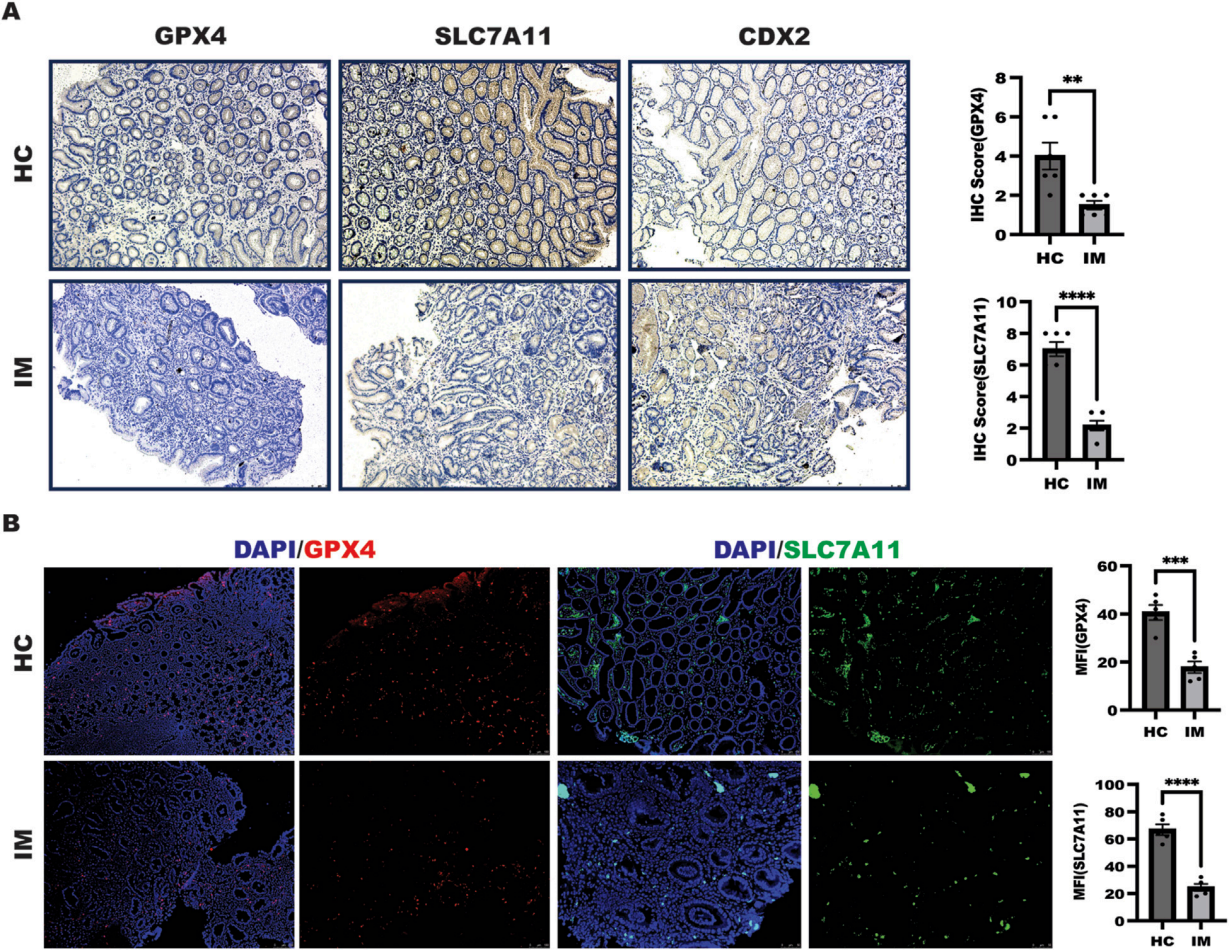
Ferroptosis was decreased in patients with IM. **(A)** IHC was performed to detect GPX4 and SLC7A11 expression as well as CDX2 expression in a set of healthy control and patients with IM, the quantitation of GPX4 and SLC7A11 expression were performed upon IHC score, **p < 0.01, ****p < 0.0001. **(B)** IF experiment was employed to determine GPX4 and SLC7A11 expression in a set of healthy control and patients with IM, the quantitation of GPX4 and SLC7A11 expression were performed upon mean fluorescence intensity (MFI) analysis, **p < 0.01, ****p < 0.0001.

The above results showed that rabeprazole suppressed gastric intestinal metaplasia through triggering ferroptosis mediated by GPX4 downregulation. To further confirm the relationship between ferroptosis and gastric intestinal metaplasia in a set of clinical samples in patients with gastric intestinal metaplasia and healthy control. As shown in Figure 5A, in comparison to healthy control (HC), a significant decreased ferroptosis characterized by enhanced GPX4 and SLC7A11 expression were observed in patients with gastric intestinal metaplasia (IM) with CDX2 expression as evidenced by IHC analysis. In line with this, the further work from IF analysis also confirmed that GPX4 and SLC7A11 expression were decreased in patients with gastric intestinal metaplasia (Figure 5B). Overall, these results implied that targeted GPX4 and SLC7A11-mediated ferroptosis is novel pathway of rabeprazole for treatment of gastric intestinal metaplasia.

## Discussion

Gastric intestinal metaplasia is a common complication for *H. pylori*-induced metaplasia-dysplasia-carcinoma sequence, which was characterized by increased CDX2 and/or MUC2 expression, which has been received PPI treatment. In this current work, we demonstrated that rabeprazole suppressed CDX2-mediated gastric intestinal metaplasia through activation of ferroptosis as evidenced by decreased GPX4 expression. Blockade of ferroptosis caused by Fer-1 in gastric epithelial cells could overcome the inhibitory effect of rabeprazole on CDX2 expression. Mechanically, rabeprazole attenuated PKA/CREB signaling, leading to inhibit phosphorylation and nuclear translocation of CREB as well as the binding ability of CREB to GPX4 promoter. Most importantly, PKA interacted with CREB, and this interaction was drastically destroyed in response to Rabeprazole stimulation. What’s more, decreased ferroptosis characterized by enhanced GPX4 and SLC7A11 expression was observed in clinical sample diagnosed with IM, suggesting ferroptosis pathway is critical for IM. Taken together, these finding greatly extended our insight into the mechanism that rabeprazole-induced ferroptosis to alleviate IM, and targeted ferroptosis with rabeprazole could be an optimal therapy strategy for patients with IM.

The work have demonostrated that the increased intestinal tight junction permeability and disease severity as well as intestinal microbiome composition alteration were observed in two independent models of DSS-induced colitis after administration of long-term PPI in mice, which might be attributed to enhanced myosin light chain kinase (MLCK) activation and expression (Nighot et al., 2023; Son et al., 2022; Pai et al., 2023). Of interest, activation of ferroptosis has been confirmed to inhibit CDX2 expression (Pan et al., 2023). Ferroptosis, an iron-dependent form of regulated cell death driven by the lethal accumulation of lipid peroxidation, has been reported to take an important role in the occurrence and development of gastric cancer, including proliferation, invasion (Lu et al., 2022; Zhao et al., 2021; Zhang et al., 2020), while limited studies were available about ferroptosis in IM. Bioinformatics analysis has revealed ferroptosis-related genes (FRGs) that may be involved in IM, such as HMOX1 and NOS gene. In this work, we presented a report that rabeprazole, an PPI therapy for patients with IM, could alleviate IM characterized by decreased CDX2 expression through enhanced GPX4-mediated ferroptosis in BGC823 cells, while no similar phenomenon was obtained in omeprazole treatment (Data unpublished). Ferroptosis suppression could rescue the inhibition of rabeprazole in CDX2 expression, which indicated rabeprazole ameliorated IM in dependent of ferroptosis. However, in addition ferroptosis, whether other cell death manner involved in the inhibitory effect of rabeprazole in IM, including cell pyroptosis, despite rabeprazole has been showed to anti-inflammatory reaction, cell proliferation or impair barrier function in gastric epithelial cells in our previous work (Yang et al., 2023; Xie et al., 2021; Zhou et al., 2021), including disulfidptosis, necroptosis, cuproptosis.

Targeted classical cAMP signaling has been reported as an important strategy in serials of disease (Du et al., 2023; Crocetti et al., 2022), which was attributed to activation of phosphodiesterase (PDE)/PKA/CREB in modulating the development of disease. For instance, targeted PDE4 by dipyridamole or roflumilast could enrich intestinal CD8^+^CD39^+^ T cells to alleviate inflammation or improve intestinal epithelial cell differentiation (Pan et al., 2023; Huang et al., 2019). Interesting, in addition to gastroparesis (McDonough et al., 2020) and acid secretion (Okuda et al., 2009) as well as gastritis, no evidences have directly confirmed the role of classical cAMP signaling in gastric IM. Despite, in this work, we showed rabeprazole could destroy PKA/CREB complex, leading to inhibit CREB nuclear translocation and phosphorylation. However. There are several issues remained to be addressed in future: (Scida et al., 2018) both phosphodiesterase (PDE) and adenylate cyclase (AC) are critical enzyme for modulating intercellular cAMP level, which could further trigger PKA/CREB phosphorylation, whether rabeprazole regulated PKA/CREB signaling through PDE/AC or neither; (Zheng et al., 2022) in addition to PKA/CREB signaling. There is other signaling pathway involved in the influence of rabeprazole in ferroptosis could be further explored, due to the fact that aging, diet and microbial metabolite were found to regulate ferroptotic stress (Du et al., 2024; Peleman et al., 2024; Wang et al., 2024), which was required to extend the clinical sample to determine the possible role of these potential factors in ferroptosis, especially the clinical sampple before and after treatment.

## Conclusion

In this work, we have showed that rabeprazole could suppress GPX4 expression to induce ferroptosis to alleviate gastric IM characterized by decreased CDX2 expression through inhibition of PKA/CREB signaling, suppling a novel insight into the mechanism of rabeprazole in gastric intestinal metaplasia treatment and revealing the targeted ferroptosis combined with rabeprazole could be an alternative therapy strategy to inhibit gastric IM.

## Data Availability

The original contributions presented in the study are included in the article/supplementary material, further inquiries can be directed to the corresponding authors.

## Abbreviations

ChIP, Chromatin Immunoprecipitation; CDX2, caudal type homeobox 2; CREB, cAMP-response element binding protein; DMEM, Dulbecco’s Modified Eagle Medium; ERK, extracellular signal-regulated kinase; Fer-1:ferrostatin-1; FBS, Fetal bovine serum; FOXF1, Forkhead Box F1; GSH: glutathione; GC, gastric cancer; GPX4, Glutathione peroxidase 4; *H. pylori*, *H. pylori*; IM: intestinal metaplasia; IF, Immunofluorescence; MUC2, mucin2; NRF2, NF-E2-related factor 2; NF-κB, nuclear factor kappa-light-chain-enhancer of activated B cells; PDE4, phosphodiesterase 4; PBS, phosphate Buffer solution; PKA, Protein kinase A; RLU, relative luciferase unit; ROS, reactive oxygen species; STAT3, Signal Transducer and Activator of Transcription 3; SDS-PAGE, sodium dodecyl sulfate polyacrylamide gel electrophoresis; SLC7A11, system Xc; WB, Western blotting; ZO-1, Zona Occludens 1.
